# Identification of the Immunological Changes Appearing in the CSF During the Early Immunosenescence Process Occurring in Multiple Sclerosis

**DOI:** 10.3389/fimmu.2021.685139

**Published:** 2021-07-12

**Authors:** Carmen Picón, Amalia Tejeda-Velarde, José Ignacio Fernández-Velasco, Manuel Comabella, Roberto Álvarez-Lafuente, Ester Quintana, Susana Sainz de la Maza, Enric Monreal, Noelia Villarrubia, José Carlos Álvarez-Cermeño, María Inmaculada Domínguez-Mozo, Lluís Ramió-Torrentà, Eulalia Rodríguez-Martín, Ernesto Roldán, Yolanda Aladro, Silvia Medina, Mercedes Espiño, Jaime Masjuan, Clara Matute-Blanch, Marta Muñoz-San Martín, Carmen Espejo, Carmen Guaza, Alfonso Muriel, Lucienne Costa-Frossard, Luisa María Villar

**Affiliations:** ^1^ Department of Immunology, Hospital Universitario Ramón y Cajal, Instituto Ramón y Cajal de Investigacón Sanitaria (IRYCIS), Red Española de Esclerosis Múltiple (REEM), Madrid, Spain; ^2^ Department of Brain Science, Imperial College London, London, United Kingdom; ^3^ Servei de Neurologia-Neuroimmunologia, Centre d’ Esclerosi Múltiple de Catalunya (Cemcat), Vall d’ Hebron Institut de Recerca, Hospital Universitari Vall d’ Hebron, Universitat Autònoma de Barcelona, Barcelona, Spain; ^4^ Department of Neurology, Hospital Clínico San Carlos, Madrid, Instituto de Investigación Sanitaria del Hospital Clínico San Carlos (IdISSC), REEM, Madrid, Spain; ^5^ Neuroimmunology and Multiple Sclerosis Unit, Department of Neurology, Hospital Dr. Josep Trueta, Institut d’Investigació Biomèdica de Girona (IDIBGI), Girona, Medical Sciences Department, Universitat de Girona, REEM, Girona, Spain; ^6^ Department of Neurology, Hospital Universitario Ramón y Cajal, IRYCIS, REEM, Madrid, Spain; ^7^ Department of Neurology, Hospital Universitario de Getafe, REEM, Madrid, Spain; ^8^ Neuroimmunology Group, Functional and Systems Neurobiology Department, Instituto Cajal, CSIC, Madrid, Spain; ^9^ Clinical Biostatistics Unit, Hospital Universitario Ramón y Cajal, IRYCIS, CIBERESP, Nursing Department, Universidad de Alcalá, Madrid, Spain

**Keywords:** multiple sclerosis, aging, innate immunity, adaptive immunity, inflammation

## Abstract

Patients with multiple sclerosis (MS) suffer with age an early immunosenescence process, which influence the treatment response and increase the risk of infections. We explored whether lipid-specific oligoclonal IgM bands (LS-OCMB) associated with highly inflammatory MS modify the immunological profile induced by age in MS. This cross-sectional study included 263 MS patients who were classified according to the presence (M+, n=72) and absence (M-, n=191) of LS-OCMB. CSF cellular subsets and molecules implicated in immunosenescence were explored. In M- patients, aging induced remarkable decreases in absolute CSF counts of CD4+ and CD8+ T lymphocytes, including Th1 and Th17 cells, and of B cells, including those secreting TNF-alpha. It also increased serum anti-CMV IgG antibody titers (indicative of immunosenescence) and CSF CHI3L1 levels (related to astrocyte activation). In contrast, M+ patients showed an age-associated increase of TIM-3 (a biomarker of T cell exhaustion) and increased values of CHI3L1, independently of age. Finally, in both groups, age induced an increase in CSF levels of PD-L1 (an inductor of T cell tolerance) and activin A (part of the senescence-associated secretome and related to inflammaging). These changes were independent of the disease duration. Finally, this resulted in augmented disability. In summary, all MS patients experience with age a modest induction of T-cell tolerance and an activation of the innate immunity, resulting in increased disability. Additionally, M- patients show clear decreases in CSF lymphocyte numbers, which could increase the risk of infections. Thus, age and immunological status are important for tailoring effective therapies in MS.

## Introduction

Multiple sclerosis (MS) is an autoimmune disease that is characterized by demyelination, chronic inflammation, and neuronal loss, causing irreversible damage to the central nervous system (CNS). Accumulating evidence suggests that aging is a risk factor for the progression of MS, and late onset of the disease (40-50 years of age) is associated with earlier conversion to progressive phases (1). Additionally, age reduces the capability of the CNS to remyelinate (2) and increases extracellular accumulation of iron (3), mitochondrial dysfunction (4), and chronic microglia activation (5).

Age-related changes in the immune system have been extensively studied (6, 7). Aging is generally considered to be associated with chronic low-grade inflammation, which affects the innate and adaptive immune systems in a phenomenon known as “immunosenescence” (8). The innate immune system increases the secretion of pro-inflammatory cytokines and proteases, including Tumor Necrosis Factor (TNF)-α, interleukin (IL)-6, and metalloproteases. This is associated with reduced tissue clearance and phagocytosis capacity by myeloid cells (9). On the other hand, the adaptive immune system displays a reduction in the number of naïve T cells due to thymic involution, which reduces their ability to react against new antigens (10).

Importantly, the number of memory T cells in the bloodstream increases, especially the CD8 subset (11). In particular, an increase in memory T cells and antibodies specific for cytomegalovirus (CMV) has been reported, which is broadly considered to be a hallmark of immunosenescence (12). Remarkably, this process that usually occurs in people older than 65 years arises about 20 years earlier in different inflammatory diseases including MS. This phenomenon is named as premature or early immunosenescence (13–15).

Investigating the influence of a highly inflammatory disease in the immunosenescence process could help to identify whether age-related changes are a uniform process in MS or depend on patient idiosyncrasies.

To explore this, we studied the effect of aging in the adaptive and innate immune responses in the CSF of MS patients. We also explored whether the intrathecal synthesis of lipid-specific oligoclonal IgM bands (LS-OCMB), a well-established marker of a high inflammatory disease course in MS (16, 17), plays a role in this process. Understanding the age-related alterations in the immune system of MS patients is critical for the development of targeted therapeutic approaches and the discovery of novel potential markers of the progression of the disease.

## Materials and Methods

### Study Approval

This study was approved by the Ethical Committee of Ramón y Cajal University Hospital (Madrid, Spain). Written informed consent was obtained from every patient before inclusion in the study.

### Patients

In this cross-sectional prospective study, we included 263 consecutive patients (149 females/114 males) who were diagnosed with MS at Ramón y Cajal University Hospital (Madrid, Spain) according to modified McDonald criteria (18). Patients did not receive any disease-modifying treatment before inclusion. Patient characteristics are shown in **Table 1**. The expanded disability status scale (EDSS) score and the MS severity score (MSSS) were evaluated at lumbar puncture, or in case the patient was in a relapse at that moment, one month after when the clinical situation was stabilized. EDSS score was measured in the whole patient cohort, MSSS in the 161 patients with more than six months of disease duration at lumbar puncture (41 M+ and 120 M-), and the number of relapses in the first year in the 119 patients with RRMS and more than six months of disease duration (34 M+ and 101 M-).

**Table 1:** Clinical and demographic data of patients included in the study.

	Total patients (n=263)	M- patients (n=191)	M+ patients (n=72)	P
Age (years). Median (range)	39.0 (16-65)	40.0 (16-65)	35.0 (18-62)	ns
Disease duration (months) (*)	7.4 (3.2-32.1)	8.4 (3.7-45.4)	6.5 (2.0-15.5)	ns
Sex (male/female)	114/149	80/111	34/38	ns
Disease onset (RR/PP)	231/32	171/20	60/12	ns
EDSS score (*)	1.5 (1.5-2.0)	1.5 (1.0-2.0)	2.0 (1.5-3.0)	0.004
MSSS score (**)	4.3 (2.4-5.9)	4.3 (2.4-5.2)	5.9 (4.3-7.9)	<0.0001
N. of Relapses in the previous year (*)(**)	1 (1-2)	1 (1-1)	2 (1-2)	<0.0001
CSF CHI3L1 (ng/ml) (*)	209.5 (159.5-284.8)	200.0 (156.0-266.2)	246.5 (183.1-395.7)	0.017
CSF NfL (pg/ml) (*)	924.4 (567.3-2058.7)	842.6 (466.0-1364.5)	1558.9 (759.7-4053.1)	0.0002
CSF C3 (ng/ml) (*)	9586 (6808-13744)	8172 (6256-10856)	10498 (8305-15151)	0.018
IgG Index (*)	0.91 (0.72-1.41)	0.91 (0.69-1.42)	0.76 (0.54-0.98)	ns
IgM Index (*)	0.16 (0.11-0.27)	0.14 (0.10-0.22)	0.24 (0.15-0.42)	<0.0001
Alb Index (*)	4.24 (3.10-5.65)	4.24 (3.03-5.43)	4.24 (3.36-5.89)	ns

(*): Data shown as Median (25-75% IQR); (**): Explored in patients with at least six months of disease duration. Alb, Albumin; CHI3L1, Chitinase 3-like 1; C3, C3 complement component; CSF, cerebrospinal fluid; EDSS, Expanded Disability Status Scale; IQR, Interquartile range; LP, lumbar puncture; M+ patients, Those showing lipid-specific oligoclonal IgM bands; M- patients, Those lacking lipid-specific oligoclonal IgM bands; MSSS, Multiple Sclerosis Severity Score; N., number; NfL, neurofilament light chain; ns, not significant; PP, Progressive onset; RR, relapsing remitting onset. P values are referred to M+ vs M- patient comparisons. Continuous variables were analyzed using Mann-Whitney U test and categorical ones by Chi-square tests.

### Samples

Paired serum and CSF samples were always obtained for clinical purposes. Fresh CSF samples were centrifuged at 500 g for 10 min. The cellular pellet was resuspended and analyzed for subsequent flow cytometry studies as described below. After centrifugation, CSF and serum samples were aliquoted and stored at -80°C until assessment.

### Immunoglobulin G and M Oligoclonal Band Detection

Serum and CFS IgG, IgM, and albumin were quantified by nephelometry in an Immage nephelometer (Beckman Coulter, Brea, CA). Oligoclonal IgG and IgM bands were studied in paired CSF and serum samples by isoelectric focusing and immunoblotting as described previously (16, 19). The presence of intrathecal IgG or IgM synthesis was demonstrated by the appearance of two or more oligoclonal bands in CSF, not present in paired serum sample. Lipid specific IgM bands were assessed in the CSF of patients showing intrathecal IgM synthesis as previously described (16). Briefly, oligoclonal IgM bands are separated by isoelectrofocusing and transferred to nitrocellulose membranes coated with different lipids and blocked with Polypep (Merck). A membrane blocked with polypep is used as negative control. The presence of anti-lipid IgM antibodies restricted to the CSF is then evidenced by immunoblotting with anti-human IgM antibodies labeled with biotin and with streptavidin labeled with alkaline phosphatase. A representative image of anti-lipid IgM bands is shown in **Supplementary Figure 1**.

### CSF Leukocyte Subpopulations

The following monoclonal antibodies were used in the study: CD14-FITC, IFN-γ-FITC, GM-CSF-PE, CD3-PerCP, TNF-α-PerCP-Cy5.5, CD16-PE-Cy7, CD19-PE-Cy7, CD56-APC, CD8-APC-H7, CD3-BV421, and CD45-V500 (BD Biosciences, San Diego, CA). IL-17-APC was obtained from R&D Systems, Minneapolis, MN. For patients whose CSF samples were 3-5 ml (36 M+ and 86 M- patients), only a tube measuring surface antigens was studied. In cases of 5-8 ml of CSF (36 M+ and 105 M- patients), the samples were divided into two identical aliquots, and surface antigens and intracellular cytokine production were studied as detailed below. The precise CSF volume used in every case was recorded to calculate total cell numbers.

### Flow Cytometry Analysis

For surface antigen identification, cellular pellets obtained after CSF centrifugation were resuspended in the residual volume (about 100 µl), stained with the appropriate amounts of monoclonal antibodies for 30 minutes at 4°C in the dark, washed twice with PBS, and analyzed in a FACSCanto II flow cytometer (BD Biosciences). For intracellular cytokine detection, cellular pellets were stimulated and stained for flow cytometry analysis as described previously (20). In brief, cellular pellets were incubated for 4 hours at 37°C in 5% CO2 with 50 ng/ml Phorbol 12-myristate 13-acetate (PMA) (Sigma-Aldrich, St. Louis, MO) and 750 ng/ml Ionomycin (Sigma-Aldrich) in presence of 2 µg/ml Brefeldin A (GolgiPlug, BD Biosciences) and 2.1 µM Monensin (Golgi Stop, BD Biosciences). After incubation, cells were washed and stained with the monoclonal antibodies recognizing the surface antigens. Then, cells were washed, fixed and permeabilized with Cytofix/Cytoperm Kit (BD Biosciences), washed twice and subjected to intracellular staining with monoclonal antibodies recognizing different cytokines. Then, cells were washed and analyzed in a FACSCanto II flow cytometer. Data analysis was performed using the software FACSDiva V.8.0 (BD Biosciences) and the gating strategy shown in **Supplementary Figures 2**, **3**. All labeled cells were acquired to calculate total cell numbers.

### Detection of Soluble Molecules in CSF

We used ELISA to explore the CSF values of the following molecules: activin A (R&D Systems, MN), chitinase 3-like 1 (CHI3L1; Quidel Corporation, San Diego, CA), C3 complement component (Abcam; Cambridge, UK), neurofilament light chains (NfL; Uman Diagnostics, Sweden), programmed death-ligand 1 (PD-L1; R&D Systems), and T-cell immunoglobulin and mucin domain 3 (TIM-3; Bio-Techne, R&D Systems). All assays were run according to the manufacturer’s instructions with the exception of NfL, for which 10 and 50 µl of CSF were assayed for every patient.

### Anti-CMV IgG ELISA

Serum anti-CMV IgG antibodies were measured by ELISA (Zeus Scientific, USA) according to the manufacturer’s instructions. The results were expressed as an index value (IV) that was calculated as follows: 10 x sample absorbance/cut-off value. Samples were analyzed in duplicate for each test.

### Statistical Analyses

Statistical analyses were done using the software GraphPad Prism 6.0 (GraphPad Prism Inc., La Jolla, CA) and Stata 16 (StataCorp LLC, Lakeway, TX). For continuous variables, we used the Mann-Whitney U-test with Bonferroni post-hoc correction or the Kruskal-Wallis test with Dunn’s multiple comparison post-test when comparing 3 or more groups. The chi-squared test was used to compare categorical variables. P-values below 0.05 were considered as significant. Spearman correlation was used to test for associations between groups, and the Spearman r and p values are reported in each instance. Multivariate regression analysis was used to explore the relationship between age and immunological factors and between age and disability scores while adjusting for disease duration.

## Results

### Patient Characteristics

We investigated the association between aging and intrathecal immune response in MS by studying a large cohort of treatment-naive MS patients (n=263). We stratified them in two groups according to the absence (M-, n=191) and presence (M+, n=72) of LS-OCMB. Clinical, demographic, and laboratory data are shown in **Table 1**. M+ patients showed higher number of relapses in the previous year (p<0.0001), and higher values of the EDSS) (p=0.004) and the MSSS (p<0.0001) scores. In addition, they showed increased CSF values of CHI3L1 (p=0.017), neurofilament light chains (NfL) (p=0.0002), C3 complement factor (p=0.018) and of the IgM index (p<0.0001).

### Aging Diminishes Numbers of Intrathecal Lymphocytes and NK Cells in M- Patients

Next, we studied age-related changes in the main leukocyte subsets in the CSF. The results are shown in **Table 2**. M- patients showed a remarkable decrease of mononuclear cell numbers (p<0.0001) due to a drop-in lymphocytes (p<0.0001) and natural killer cells (NK) (p=0.0001). A similar reduction was found in CD4+ (p<0.0001) and CD8+ (p<0.0001) T cells and in B cells (p<0.0001). In contrast, monocyte numbers did not show any significant changes (**Table 2**). We also explored the intracellular production of pro-inflammatory cytokines by CSF lymphocytes. In the M- group, age induced significant decreases of CD4+ and CD8+ T cells producing IFN-*γ*, TNF-α, and GM-CSF, as well as CD4+ T cells producing IL-17. A reduction in B cells producing TNF-α was observed in both groups of patients (**Table 2**).

**Table 2:** Correlations between age and variables related to immune response or axonal damage.

	Total patients (n=263)		M- patients (n=191)		M+ patients (n=72)	
Variable	r	p	r	p	r	p
Leukocyte subsets (cells/ml)						
Total Mononuclear cells	-0.40	<0.0001	-0.46	<0.0001	0.02	0.886
Total Lymphocytes	-0.40	<0.0001	-0.47	<0.0001	0.02	0.889
CD4+ T cells	-0.37	<0.0001	-0.45	<0.0001	0.07	0.597
CD4+T cells IFN-gamma+	-0.31	0.0002	-0.33	0.0007	-0.15	0.364
CD4+T cells TNF-alpha+	-0.32	0.0001	-0.35	0.0003	-0.14	0.411
CD4+T cells IL-17+	-0.34	<0.0001	-0.31	0.0012	-0.34	0.042
CD4+ GM-CSF+	-0.41	0.002	-0.44	0.003	-0.28	0.378
CD8+ T cells	-0.34	<0.0001	-0.41	<0.0001	0.02	0.852
CD8+T cells IFN-gamma+	-0.30	0.0003	-0.32	0.0008	-0.11	0.510
CD8+T cells TNF-alpha+	-0.27	0.0014	-0.29	0.003	-0.08	0.614
CD8+T cells IL-17+	-0.21	0.0136	-0.15	0.121	-0.23	0.172
CD8+ GM-CSF+	-0.43	0.001	-0.49	0.001	-0.23	0.477
CD19+ B cells	-0.41	<0.0001	-0.49	<0.0001	0.02	0.847
CD19+ TNF-alpha+	-0.41	0.002	-0.36	0.02	-0.68	0.01
CD19+ GM-CSF+	-0.31	0.02	-0.25	0.103	-0.65	0.02
Total NK cells	-0.31	0.0002	-0.39	0.0001	-0.08	0.590
Total Monocytes	-0.09	0.142	-0.13	0.070	0.03	0.795
Soluble factors						
PD-L1 (pg/ml)	0.34	<0.0001	0.30	0.0011	0.56	0.0005
TIM-3 (pg/ml)	0.30	0.0012	0.27	0.04	0.41	0.002
IgG anti-CMV (IV)	0.28	0.0003	0.31	0.0006	0.17	0.247
CHI3L1 (ng/ml)	0.41	<0.0001	0.49	<0.0001	0.29	0.057
Activin A (pg/ml)	0.46	<0.0001	0.47	<0.0001	0.44	0.003
NfL (pg/ml)	-0.07	0.368	0.06	0.518	-0.28	0.051
C3 (ng/ml)	0.18	0.098	0.18	0.220	0.27	0.085

C3, C3 complement component; CHI3L1, Chitinase 3-like 1; CMV, Cytomegalovirus; CSF, Cerebrospinal fluid; GM-CSF, Granulocyte/macrophage-colony stimulating factor; IV, Index Value; NK, Natural Killer; IFN, Interferon; PD-L1, Programmed Death-ligand 1; TIM-3, T-cell immunoglobulin and mucin domain-3; TNF, Tumor necrosis factor; IL, Interleukin; M+ patients, those showing lipid-specific oligoclonal IgM bands; M- patients, those lacking lipid-specific oligoclonal IgM bands. All variables were quantified in CSF with the exception of IgG anti-CMV, quantified in serum. r and p values were determined by Spearman correlation.

**Figure 1:**
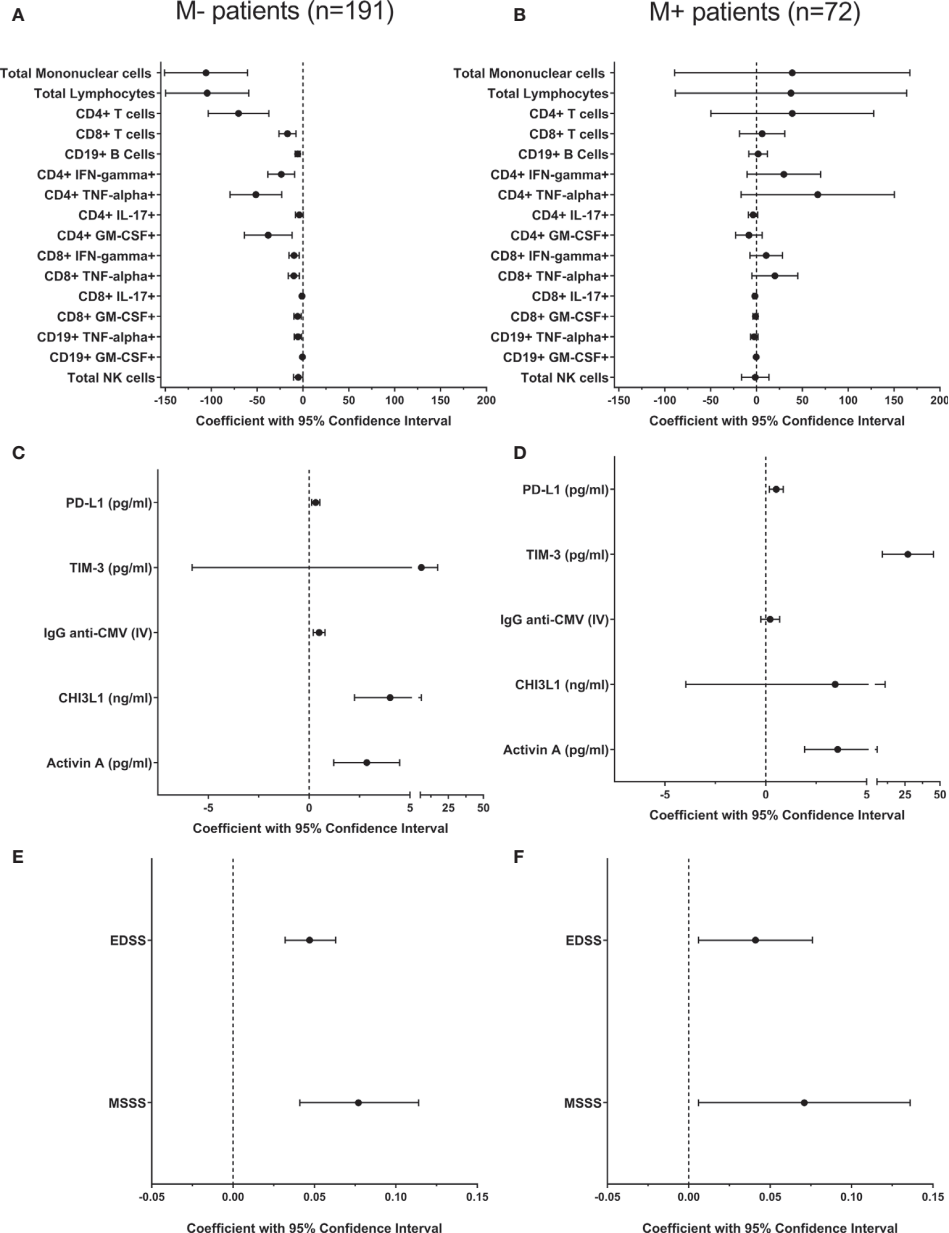
Multivariate regression analysis to explore the effect of age on immune response and disability in M- and M+ patients. We explored influence of age in different leukocyte subsets [**(A, B)** cells/ml] and in soluble factors levels **(C, D)** related to adaptive and innate immune responses in patients lacking [M-, n=191 **(A, C)**] and showing [M+, n=72, B, **(D)**] lipid-specific oligoclonal IgM bands. All variables were quantified in CSF with the exception of IgG anti-CMV, quantified in serum. The effect of age on disability in M- **(E)** and M+ **(F)** patients was also studied. All coefficients (black circles) and 95% confidence intervals were adjusted by disease duration. CHI3L1, Chitinase 3-like 1; CMV, Cytomegalovirus; CSF, cerebrospinal fluid; EDSS, Expanded Disability Status Scale; IFN, Interferon; IL, Interleukin; IV, Index Value; GM-CSF, Granulocyte/macrophage-colony stimulating factor; MSSS, MSSS, Multiple Sclerosis Severity Score. NK, Natural Killer; PD-L1, Programmed Death-ligand 1; TIM-3, T-cell immunoglobulin and mucin domain-3; TNF, Tumor necrosis factor.

To rule out the effect of disease duration on these correlations, we performed a multivariable linear regression analysis between age and leukocyte numbers adjusting for disease duration. Most associations remained significant with the only exceptions of CD4+ T cells producing IL-17 in M- patients and B cells producing TNF-α in M+ patients that were lost (**Figures 1A, B** and **Supplementary Table 1**).

**Figure 2:**
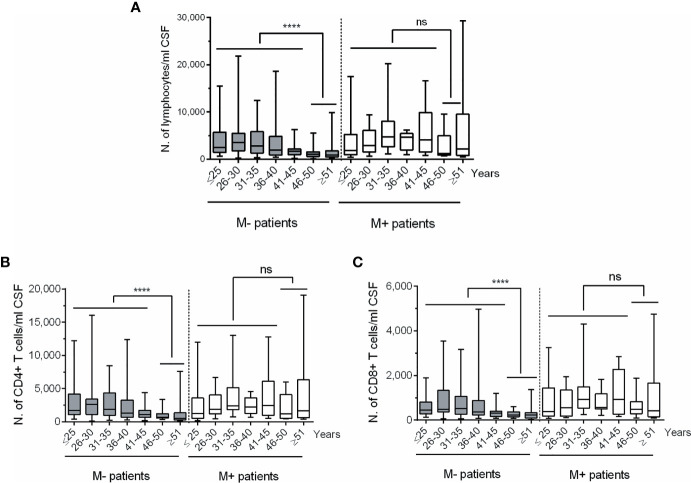
Changes in CSF lymphocyte counts in MS patients classified according to age and to the absence or presence of lipid-specific oligoclonal IgM bands (LS-OCMB). Absolute cell numbers (N.) of CSF total lymphocytes **(A)**, CD4+ **(B)** and CD8+ **(C)** T lymphocytes were studied in multiple sclerosis patients lacking (M-, grey bars, n=191) and showing (M+, white bars, n=72) LS-OCMB, classified according to their age: ≤25, 26-30, 31-35, 36-40, 41-45, 46-50 and ≥ 51 years. ns, not significant. ****p < 0.0001.

To investigate the age at which intrathecal leukocyte decline occurs, we classified patients according to their age in subgroups of five years (i.e., ≤25, 26-30, 31-35 years, and so on). The highest changes in lymphocyte numbers were observed in M- patients older than 45 years (**Figure 2A**). The same results were observed when studied CSF CD4+ and CD8+ T cell numbers (**Figures 2B, C**). Remarkably, no differences were observed in the M+ group, even in patients older than 50 years.

**Figure 3:**
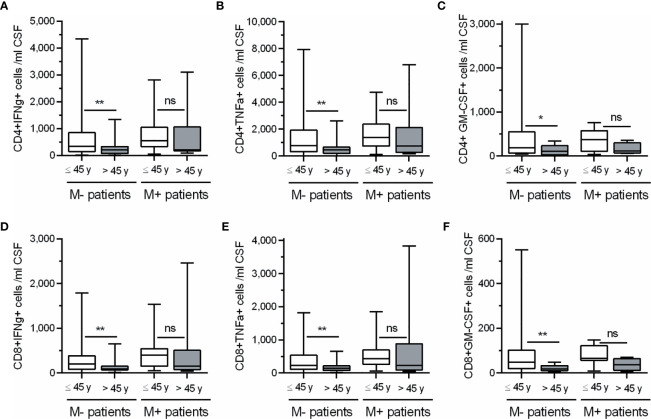
Changes in CSF CD4+ and CD8+ T lymphocytes producing cytokines in M- and M+ patients according to age. Absolute cell numbers (N.) of CD4+ **(A–C)** and CD8+ **(D–F)** T lymphocytes producing Interferon-gamma (IFNg), Tumor Necrosis Factor-alpha (TNFa) and Granulocyte-Macrophage Colony Stimulating Factor (GM-CSF) in patients lacking (M-, n=105) and showing (M+, n=36) lipid-specific oligoclonal IgM bands, and classified according to their age in ≤ 45 years (white bars) and > 45 years (grey bars). ns, not significant; y, years. *p < 0.05; **p < 0.01.

**Figure 4:**
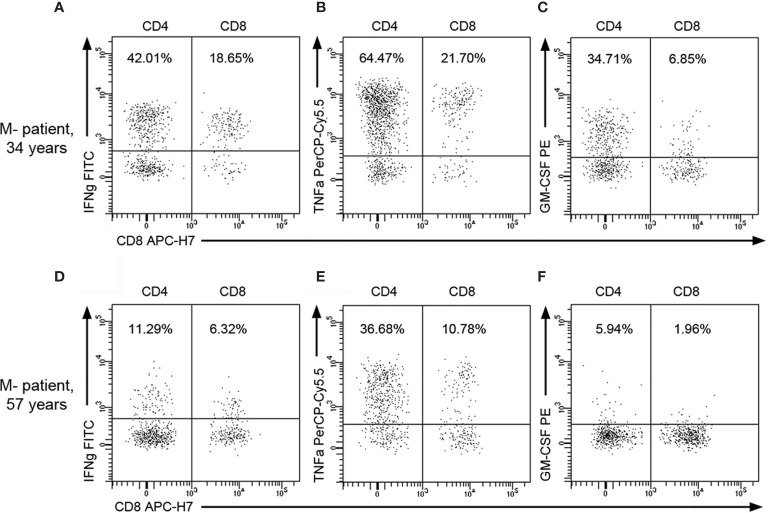
Representative flow cytometry images of CSF T lymphocytes producing cytokines in M- patients. Representative dot plots showing intracellular cytokine production by CSF CD4+ and CD8+ T lymphocytes from a young M- patient [34 years old; **(A–C)**] and from an old one [57 years; **(D–F)**]. Percentages over total CSF CD45+ lymphocytes are indicated on plots. GM-CSF, Granulocyte-Macrophage Colony Stimulating Factor; IFNg, Interferon-gamma; TNFa, Tumor necrosis factor-alpha.

In view of these results, we classified M+ and M- patients in two groups (Age ≤45 or > 45 years) and further explored the influence of age in the numbers of CD4+ and CD8+ T cells producing IFN-gamma, TNF-alpha, and GM-CSF (**Figure 3**). M- patients older than 45 years showed lower CSF numbers of CD4 and CD8 T cells producing IFN-gamma (p=0.004 and p=0.003 respectively), TNF-alpha (p=0.002 and p=0.004 respectively), and GM-CSF (p=0.029 and 0.005 respectively). However, none of these T cell subsets changed in M+ patients with age. Representative images of intracellular cytokine production by CD4+ and CD8+ T lymphocytes from M- patients are shown in **Figure 4**.

### Changes in Soluble Factors Associated With Immunosenescence and T Cell Exhaustion

To gain insight into the regulation of adaptive immune cell activity, we analyzed CSF levels of PD-L1 and TIM-3, which are markers of T cell tolerance and exhaustion, respectively. We also explored serum titers of anti-CMV IgG, a marker of immunosenescence. PD-L1 and TIM-3 levels increased with age in M- and M+ patients (**Table 2**). The association with TIM-3 was lost in M- patients after adjusting for disease duration (**Figures 1C, D** and **Supplementary Table 1**). By contrast, serum anti-CMV IgG, a marker of immunosenescence, increased with age only in the M- group (**Figures 1C, D** and **Supplementary Table 1**).

### Soluble Factors Related to Innate Immune Response

To further examine age-related differences between M+ and M- patients, we assessed CSF values of CHI3L1 and activin A, which are soluble factors related to the innate response. Activin A increased with age in both M- and M+ patients, while CHI3L1 levels only increased in the M- group. Notably, M+ patients had higher levels of this protein independently of age (**Table 1**). No significant correlations were lost after adjusting for disease duration (**Figures 1C, D** and **Supplementary Table 1**). By contrast, CSF NfL and C3 complement component levels did not change with age in M- or M+ patients (**Table 2**), but were higher in M+ patients independently of age (**Table 1**).

### Age and Disability Progression

Finally, we explored the EDSS and MSSS scores. The first variable was studied in the total patient cohort, and the second one was studied in the 161 patients with at least six months of disease duration at lumbar puncture (120 M- and 41 M+). As reported above, M+ patients showed higher values of EDSS and MSSS scores (**Table 1**). When exploring the effect of age after adjusting for disease duration, we found increases in both scores in M- and M+ patients (**Figures 1E, F** and **Supplementary Table 1**).

## Discussion

Immunosenescence is characterized by a chronic activation of the innate immune response and reduced effectiveness of the adaptive response, which commonly occur after the age of 65 years (6). Converging evidence suggests that this process can occur earlier in patients with chronic immune-system activation, such as those with acquired immunodeficiency syndrome or rheumatoid arthritis (21, 22). In MS, older age affects the response to treatments and increases the risk of side effects (23, 24).

Pathological studies involving patient necropsies have provided recent reports on the molecular mechanisms involved in these changes came (25). These studies found an increase in innate immune-cell activation restricted to the CNS and a decrease in lymphocyte influx into the CNS in progressive forms of the disease (26). However, MS is a heterogeneous disease, and this process could not be uniform in all patients. Along these lines, it was shown that LS-OCMB (which is associated with a worse course of MS (16, 26) and higher inflammation demonstrated by augmented NfL values (27) have a protective effect on PML risk in patients receiving natalizumab treatment, independently of age (17, 28). This could imply that M+ status can modulate the effect of age in MS.

We aimed to explore this idea in 263 consecutive MS patients who had not previously received any disease-modifying treatment. M- patients showed a remarkable reduction in the numbers of B, T, and NK cells in the CSF, which was not observed in M+ patients. M- patients also showed a clear reduction in the numbers of B and T cells producing pro-inflammatory cytokines. These data are interesting since they show that highly inflammatory MS can counteract the effect of age in the inflammation of the adaptive immune system.

To explore the origin of these differences, we studied soluble factors associated with immune cell exhaustion and immunosenescence. All MS patients showed a modest age-associated increase of PD-L1, a check point molecule capable of inducing T cell tolerization that rises in old individuals (29). This confirms the appearance of an early immunosenescence process in MS patients, since this study was performed in individuals aged between 16 and 65 years, an age at which most healthy individuals did not enter in the immunosenescence process yet. In addition, age induced in M+ patients an elevation in the CSF values of TIM-3, a co-receptor that frequently increases upon repeated T cell activation (30–33). TIM- favors T cell exhaustion in junction with other factors associated with senescence but its expression alone does not seem to correlate with this condition (34). Despite the increases of CSF levels of PD-L1 and TIM-3, M+ patients did not experience a decrease in the CSF levels the numbers of pro-inflammatory lymphocytes with age. This may be occasioned by the lack of other molecules involved in the immunosenescence process, or by the up-regulation of other pro-inflammatory signals. In this line, we described the association of the M+ status with polymorphisms in the TNF-alpha promoter associated with sustained inflammation (35).

Moreover, M- but not M+ patients showed an age-related increase of the titers of anti CMV antibodies. The increase of clonally expanded CD8 T cells and of anti-CMV antibodies associate with immunosenescence (12). In addition, CMV infection occurs more frequently in older individuals in MS and associates with lower production of pro-inflammatory cytokines and (36, 37). These data show that M- patients show an early immunosenescence process associated with a down-regulation of the pro-inflammatory adaptive immune response. The M- phenotype is twice as frequent as the M+ phenotype in MS. Thus, the reduction of pro-inflammatory adaptive immune cells shown by these patients may account for the changes in adaptive immune response described for aged MS patients (38), and M+ status may protect from opportunistic infections in older patients (39, 40).

Regarding the innate immune response, M+ patients showed higher values of the C3 component of the complement and of CHI3L1 in CSF, independently of age. IgM antibodies can fix complement much more efficiently than IgG antibodies, so M+ patients can have an early activation of the innate immune system mediated by the complement activation. CHI3L1 is expressed in demyelinated lesions by activated astrocytes and is related to the innate immune response (41). This may account for the early worsening of disability shown by these patients.

The M- group only showed an increase in CHI3L1 levels with aging, which suggests an additional activation of the innate immune system occurring with age. This was confirmed by the age-associated increase of activin A experienced by M+ and M- patients. This molecule is expressed by activated microglia (42), which is an important part of the senescence-associated secretoma, which induces inflammaging in older individuals (43). This indicates that MS patients enter an inflammaging process at a much younger age than healthy individuals. This could be at least partly responsible for the increased worsening of disability shown by MS patients at ages beyond 45 years (44). In view of these results, activin A levels could be a biomarker of early inflammaging in MS. Further exploration of the cytokine profile of innate immune cells in the CSF of older MS patients and its association with disability could provide tools for identifying new effective treatments for older MS patients.

## Data Availability Statement

Anonymized data supporting the findings of this study will be shared by reasonable request from any qualified investigator during three years after the publication of the study.

## Ethics Statement

The studies involving human participants were reviewed and approved by Ethical Committee of Ramón y Cajal University Hospital (Madrid, Spain). The patients/participants provided their written informed consent to participate in this study.

## Author Contributions

CP, AT-V, JF-V, SM, and NV collected CSF samples and performed the flow cytometry and ELISA experiments. CP, AT-V, NV, and LV drafted the manuscript. ER and ER-M supervised flow cytometry studies. MC and CM-B quantified CHI3L1 in CSF samples. EQ, MM-S, and LR-T contributed by sending samples. RÁ-L and MD-M performed IgG anti-CMV quantification. SSM, EM, LC-F, JÁ-C, JM, and YA visited MS patients and collected clinical data. CE and CG contributed to study the innate immune system. LV designed and supervised the study. All authors contributed to the article and approved the submitted version.

## Funding

This work was supported by grants FIS-PI15/00513, FIS-PI18/00572 and RD16/0015/0001 from the Instituto de Salud Carlos III. Ministerio de Ciencia e Innovación, Spain and FEDER: "Una manera de hacer Europa".

## Conflict of Interest

The authors declare that the research was conducted in the absence of any commercial or financial relationships that could be construed as a potential conflict of interest.
