# Supplementary material for: Identification of the Immunological Changes Appearing in the CSF During the Early Immunosenescence Process Occurring in Multiple Sclerosis
Source: Front Immunol. 2021 Jul 12;12:685139. doi: 10.3389/fimmu.2021.685139 (PMC8311928; doi:10.3389/fimmu.2021.685139)
Supplement: Supplementary file 1 [file Image_1.pdf]

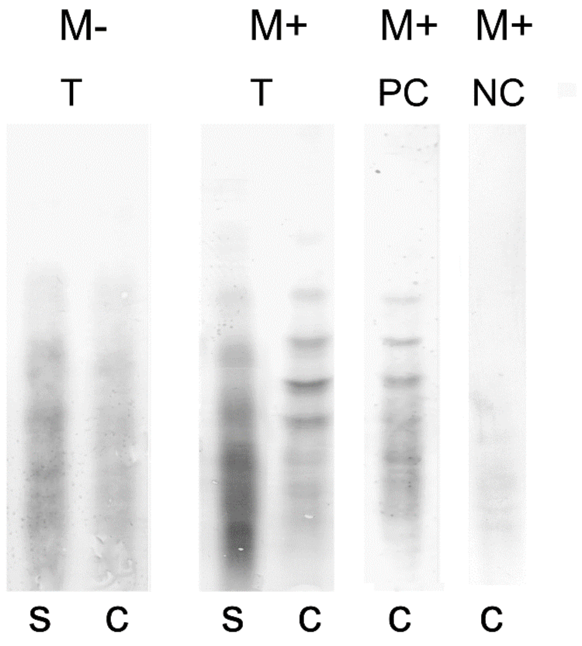

Supplementary Figure 1. **Representative images of Oligoclonal IgM Bands Detection (OCMB) by isoelectrofocusing**.

Representative immunoblots of serum (S) and cerebrospinal fluid (C) samples from two MS patients, the first one lacking (M-) and the second one showing (M+) OCMB restricted to cerebrospinal fluid. After focusing, proteins were transferred to an untreated membrane to detect total IgM bands (T), to a membrane coated with phosphatidylcholine (PC) and blocked with a mixture of peptides, and to a membrane coated with the same mixture of peptides (Negative control, NC). IgM bands were detected by incubating membranes with an anti-human IgM antibody labeled with biotin followed by incubation with alkaline phosphatase-labeled streptavidin. Anti-PC OCMB were detected in the PC-coated membrane and not in the negative control.
