# Supplementary material for: Identification of the Immunological Changes Appearing in the CSF During the Early Immunosenescence Process Occurring in Multiple Sclerosis
Source: Front Immunol. 2021 Jul 12;12:685139. doi: 10.3389/fimmu.2021.685139 (PMC8311928; doi:10.3389/fimmu.2021.685139)
Supplement: Supplementary file 2 [file Image_2.pdf]

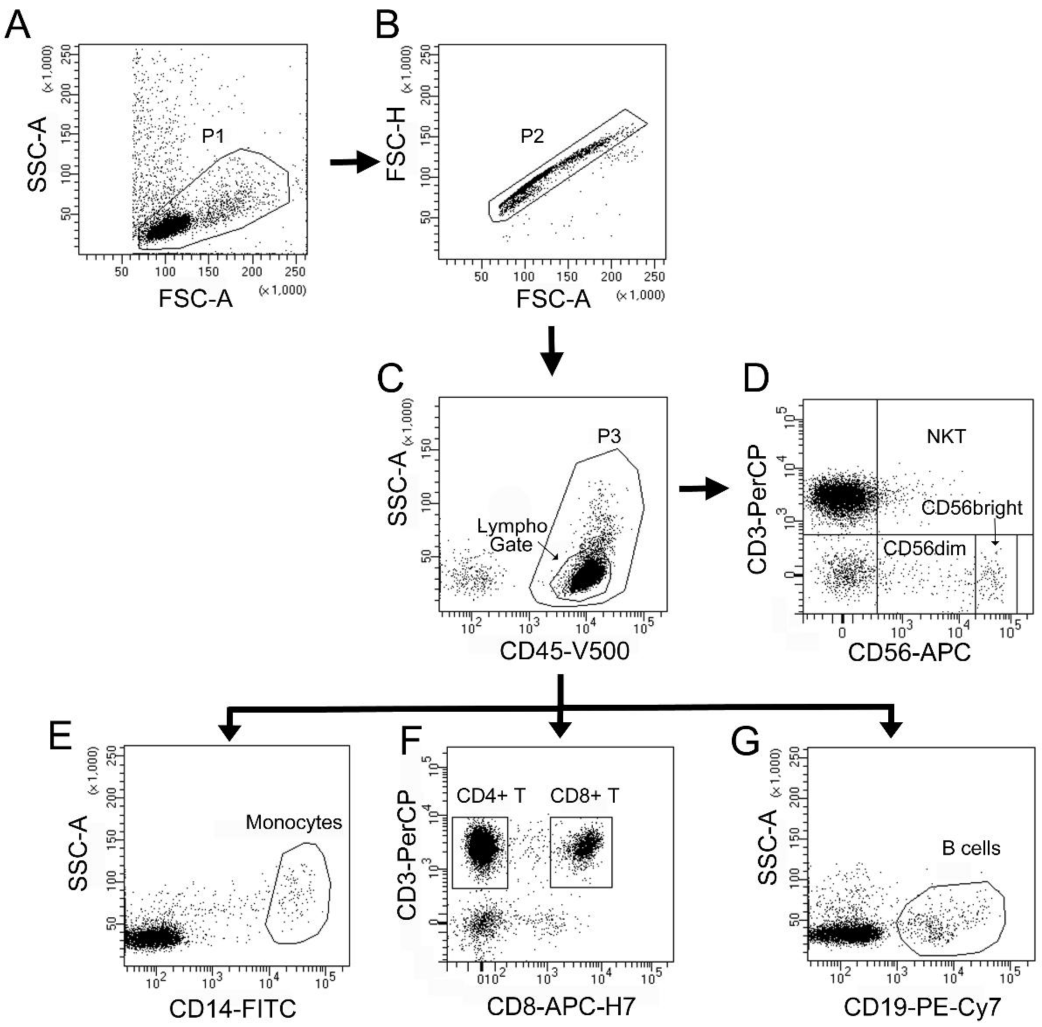

Supplementary Figure 2. **Gating strategy employed for the identification of CSF leukocyte subsets by flow cytometry**.

An initial region was set on the Forward-Area (FSC-A) vs Side-Area (SSC-A) scatter dot plot to exclude debris or apoptotic cells (panel A, gate P1), and then a second region was drawn on the FSC-A vs FSC-Height (FSC-H) scatter to exclude duplets (panel B, gate P2). A third region was set around cells expressing intermediate to high CD45 with low to intermediate side scatter to select total mononuclear cells (panel C, gate P3) and total lymphocytes (panel C, Lympho Gate). Fluorescence emitted from cells in gate P3 were further analyzed. Thus, cells expressing CD56+ (panel D) were subdivided into CD3- CD56dim cells (gate CD56dim), CD3+ CD56dim (gate NKT) and CD3- CD56hi (gate

CD56bright). Total monocytes were identified as cells expressing CD14hi with intermediate SSC (panel E). CD3+ T cells (panel F) were classified as CD4+ T cells (CD3+ CD8-) and CD8+ T cells (CD3+ CD8hi). B lymphocytes were identified as CD19+ cells (panel G).
