# Supplementary material for: Identification of the Immunological Changes Appearing in the CSF During the Early Immunosenescence Process Occurring in Multiple Sclerosis
Source: Front Immunol. 2021 Jul 12;12:685139. doi: 10.3389/fimmu.2021.685139 (PMC8311928; doi:10.3389/fimmu.2021.685139)
Supplement: Supplementary file 3 [file Image_3.pdf]

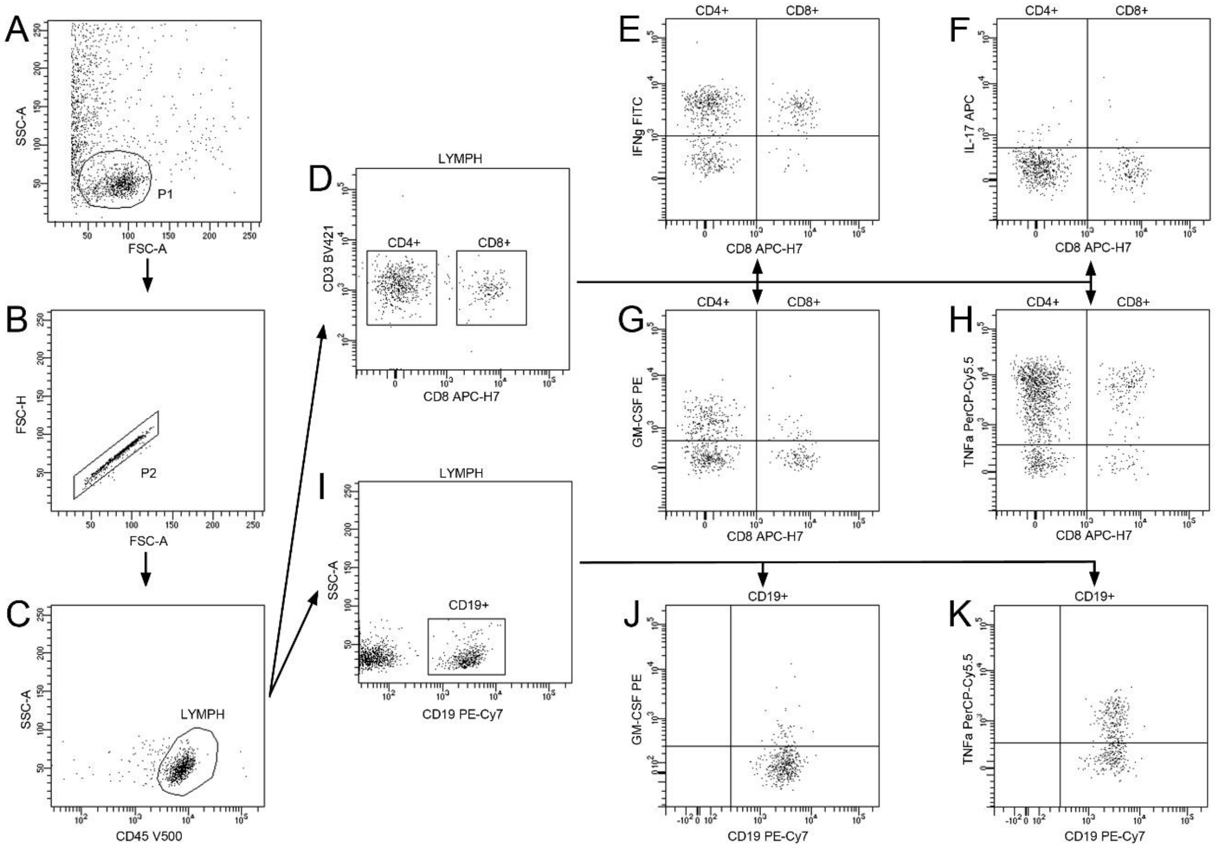

Supplementary Figure 3. **Gating strategy employed for the identification of CSF lymphocyte subsets producing cytokines by flow cytometry**.

Total events were first gated to exclude debris and apoptotic cells (A, gate P1) and then gated for doublet discrimination (B, gate P2). Cells were further analyzed to identify lymphocytes (C, gate LYMPH) by their side scatter area (SSC-A) and CD45 staining. Expression of CD3 and CD8 identified total CD4+ (D, gate CD4+) and CD8+ (D, gate CD8+) T cells. According to their intracellular cytokine staining, we identified CD4+ and CD8+ T cells producing interferon-gamma (IFNg, E), interleukin-(IL)-17 (F), granulocyte macrophage colony-stimulating factor (GM-CSF, G) and tumor necrosis factor-alpha (TNFa, H). B cells were identified by their CD19 expression (I, gate CD19+) and gated for GM-CSF (J) and TNFa (K) production.
