# Supplementary material for: Identification of the Immunological Changes Appearing in the CSF During the Early Immunosenescence Process Occurring in Multiple Sclerosis
Source: Front Immunol. 2021 Jul 12;12:685139. doi: 10.3389/fimmu.2021.685139 (PMC8311928; doi:10.3389/fimmu.2021.685139)
Supplement: Supplementary file 4 [file Table_1.docx]

**Supplementary Table 1:** Multivariate regression analysis to explore the effect of age on adaptive and innate immune responses and on disability of M- and M+ patients.

|  | M- patients (n=191) | | M+ patients (n=72) | |
| --- | --- | --- | --- | --- |
| Variable | Coef | 95% CI | Coef | 95% CI |
| CSF leukocyte subsets (cells/ml) |  |  |  |  |
| Total Mononuclear cells | -105.69 | (-150.93 ;-60.46) | 38.96 | (-89.30 ;167.22) |
| Total Lymphocytes | -104.44 | (-149.75 ;-59.13) | 37.59 | (-88.58 ;163.76) |
| CD4+ T cells | -70.26 | (-103.38 ;-37.14) | 39.02 | (-49.70 ;127.73) |
| CD4+ IFN-gamma+ | -23.69 | (-38.31 ;-9.07) | 29.81 | (-10.25 ;69.87) |
| CD4+ TNF-alpha+ | -51.31 | (-79.49 ;-23.14) | 66.79 | (-16.75 ;150.34) |
| CD4+ IL-17+ | -4.05 | (-8.31 ;0.21) | -3.78 | (-8.85 ;1.28) |
| CD4+ GM-CSF+ | -37.92 | (-63.91 ;-11.94) | -8.30 | (-22.79 ;6.18) |
| CD8+ T cells | -16.88 | (-26.14 ;-7.62) | 6.14 | (-18.52 ;30.80) |
| CD8+ IFN-gamma+ | -9.70 | (-15.30 ;-4.11) | 10.59 | (-7.14 ;28.33) |
| CD8+ TNF-alpha+ | -10.00 | (-15.98 ;-4.01) | 20.01 | (-5.03 ;45.04) |
| CD8+ IL-17+ | -1.05 | (-2.46 ;0.35) | -1.77 | (-3.64 ;0.10) |
| CD8+ GM-CSF+ | -5.89 | (-9.85 ;-1.92) | -1.09 | (-3.82 ;1.63) |
| CD19+ B Cells | -5.77 | (-8.48 ;-3.06) | 1.76 | (-8.41 ;11.94) |
| CD19+ TNF-alpha+ | -5.55 | (-9.54 ;-1.56) | -2.49 | (-6.56 ;1.59) |
| CD19+ GM-CSF+ | -0.63 | (-1.26 ;-0.00) | -0.38 | (-1.29 ;0.52) |
| Total NK cells (cells/ml) | -5.16 | ( -10.21 ; -0.12) | -1.40 | ( -16.33; 13.54) |
| Soluble factors |  |  |  |  |
| PD-L1 (pg/ml) | 0.33 | (0.13 ;0.53) | 0.52 | (0.18 ;0.87) |
| TIM- 3 (pg/ml) | 5.80 | (-5.80 ;17.40) | 27.13 | (8.91 ;45.35) |
| Serum IgG anti-CMV (IV) | 0.50 | (0.21 ;0.79) | 0.22 | (-0.24 ;0.69) |
| CHI3L1 (ng/ml) | 4.02 | (2.25 ;5.79) | 3.44 | (-3.96 ;10.84) |
| Activin A (pg/ml) | 2.86 | (1.22 ;4.49) | 3.57 | (1.93 ;5.20) |
| Disability scores |  |  |  |  |
| EDSS | 0.05 | (0.03 ;0.06) | 0.04 | (0.006 ;0.08) |
| MSSS | 0.08 | (0.04 ;0.11) | 0.07 | (0.006 ; 0.14) |

Footnote: CHI3L1: Chitinase 3-like 1; Coef: Coefficient; CI: Confidence Interval; CMV: Cytomegalovirus; CSF: cerebrospinal fluid; EDSS: Expanded Disability Status Scale; IFN: Interferon; IL: Interleukin; IV: Index Value; GM-CSF: Granulocyte/macrophage-colony stimulating factor; NK: Natural Killer. M+ patients: Those showing lipid-specific oligoclonal IgM bands; M- patients: Those lacking lipid-specific oligoclonal IgM bands; MSSS: Multiple Sclerosis Severity Score; PD-L1: Programmed Death-ligand 1; TIM-3: T-cell immunoglobulin and mucin domain-3; TNF: Tumor necrosis factor. All soluble factors were quantified in CSF with the exception of IgG anti-CMV, quantified in serum. All Coefficients and Confidence Intervals were adjusted by disease duration.
